# Farm households' flood adaptation practices, resilience and food security in the Upper East region, Ghana

**DOI:** 10.1016/j.heliyon.2020.e04167

**Published:** 2020-06-11

**Authors:** Hamdiyah Alhassan

**Affiliations:** Dept. of Agricultural and Resource Economics, University for Development Studies

**Keywords:** Non-farm, Multinomial endogenous treatment effect, Flood adaptation, Food security, Ghana, Agricultural economics, Econometrics, Environmental economics, Food economics, Poverty, Economics, Agriculture, Environmental science

## Abstract

This study employs the multinomial endogenous treatment effect model to examine the effect of flood adaptation strategies on farm households' food security in the Upper East region, Ghana. In addition, an ordered probit model was used to analyse the determinants of household's recovery from flood shocks. Farmers adopt on-farm and non-farm activities as adaptation strategies. Estimation results indicate that farmers that employ on-farm and non-farm strategies had their food security situation improved and recovered faster from flood shocks. Age, education, access to extension, credit, farm size and information on flood occurrence drive the farmer's decision to adopt on-farm practices. Marital status, education, farm size and information on flood occurrence significantly influenced adaptation decisions related to non-farm activities. Other factors that influence household's recovery period from flood events were age, education, FBO and perceived severity of flood. Programs and policies that promote extension contacts, increase awareness on flood occurrences and provide non-farm work opportunities can be beneficial to reduce the adverse effects of floods.

## 1 Introduction

The adverse effects of flood events have been widely recognized and documented by policymakers and researchers. It is well known that, flood events induce risks in most agrarian economies because of their dependence on rain-fed agriculture. Further, the challenges of widespread poverty, poor infrastructure development and weak adaptive capacity make these economies highly vulnerable to flood events (Intergovernmental Panel on Climate Change (IPCC), 2014). Moreover, projected increases in extreme climate events, are also expected to have deleterious impact on the agriculture sector (Adenle et al., 2017; IPCC, 2014), that supports many households across sub-Saharan Africa (SSA). Evidence points to the fact that climate-related shocks such as floods have significant negative effect on crop yield, food security and the economy (Amouzou et al., 2019; Abrams et al., 2018). It has been reported that, in Africa extreme climate events can reduce the length of growing seasons; which could result in decrease crop yields estimated at 20–50 percent by 2050 (Connolly-Boutin and Smit, 2015; Sarr, 2012).

Recognizing the negative effects of flood events in the agriculture sector, policy makers and researchers have devoted themselves to reduce the adverse effect of flood disasters on agriculture. Subsequently, empirical studies have been conducted to identify farm households' adaptation strategies to flood events. Growing literature showed that farm households' adapt by adopting on-farm and non-farm strategies. Adjustment in sowing time, use of drought tolerant varieties, crop diversification, crop rotation and shifting to new crops are some of the on-farm practices adopted by farmers (Thennakoon et al., 2020; Ali and Rahut, 2020; Alhassan et al., 2019). Farmers' also engaged in non-farm activities and migrate to enable them adapt to flood events (Iqbal et al., 2015; Abid et al., 2016). The concern of most policymakers is, whether these adaptation strategies help build the resilience of households to climate-related shocks. Resilience to climate-related shock describes the capacity of a farm household to absorb climate stresses and adjust without worsening its functions and structure (Walker et al., 2004). Thus, resilience is very critical for sustainability in the face of increasingly consistent and unpredictable climate shocks. However, the adaptation strategies used during flood events are influenced by many factors. Available empirical literature showed that farmers' socio-economic characteristics, farm-level and institutional factors influence their response to flood events (Thennakoon et al., 2020; Ali and Rahut, 2020; Deressa et al., 2009) which is area or country specific. It was against these backdrops that, this study examines the factors that influence adaptation decisions, as well as the effect of flood adaptation strategies on farm households' food security and their recovery from flood shocks in Upper East region of Ghana.

Ghana, like most developing countries has larger proportion of its labour force employed in the agriculture sector. Agricultural activities are mainly rain-fed making it highly vulnerable to extreme weather conditions such as high temperature, erratic rainfall and floods (Yiran and Stringler, 2016). However, Ghana's savannah ecological zone carries a higher proportion of the burden of climatic hazards. This is because the savannah ecological zone is highly vulnerable to climate change (Armah et al., 2010) and is already struggling with a higher incidence of poverty and food insecurity (Ghana Statistical Service (GSS, 2013)). In recent years, the frequencies and incidences of floods have been increasing in the Upper East region of Ghana. The largest floods occurred in 2007, 2010, 2012, 2017, 2018 and 2019 which was attributed to heavy rains and the opening of the flood gate of the Bagre dam by Burkina Faso (Fiasorgbor et al., 2018; Yiran and Stringler, 2016). These flood events pose significant threats to agricultural production, and rural livelihoods. Studies have revealed that floods causes crop loss, loss of livestocks, destruction of houses, injury and death of people (Fiasorgbor et al., 2018; Yiran and Stringler, 2016). The adverse impacts of flood events on the agriculture sector have resulted into perpetual poverty and food insecurity in the Upper East region (Yiran and Stringler, 2016). As a step towards improving farmer's welfare, it is necessary to have knowledge on the determinants of adaptation decisions and it effect on food security and flood recovery since such studies are scarce.

Most of the empirical studies focused generally on climate change adaptation strategies, it drivers and welfare effects (see Shahzad and Abdulai, 2020; Alhassan et al., 2019; Abid et al., 2016; Ali and Erenstein, 2017; Di Falco et al., 2011). Growing literature point to the fact that, adaptation to climate change improves farm productivity and income (Shahzad and Abdulai, 2020; Ali and Erenstein, 2017; Abid et al., 2016). Moreover empirical studies on farmers' adaptation decisions, compare the welfare measures between adapters and non-adapters without categorising the adaptation strategies into on-farm and non-farm strategies (see Shahzad and Abdulai, 2020; Di Falco et al., 2011). Adopting on-farm strategies reduce crop loss, and thus enhance farm household income and food security (Shahzad and Abdulai, 2020). However, engagements in non-farm activities have conflicting effects. According to the lost-labour hypothesis, reallocation of resources and labour from on-farm to non-farm activities reduces agricultural productivity and thus household's food security (Dedehouanou et al., 2018; Omiti et al., 2009). On another hand, if non-farm income is used to purchase improved farm inputs it enhances farm productivity, income and food security (i.e. the liquidity-relaxing effect) (Dedehouanou et al., 2018). Moreover, an empirical examination of this claim is scarce in the literature.

Motivated by these observations, the present study extends the literature further to analyse the effect of on-farm and non-farm flood adaptation strategies on farm households' food security using the multinomial endogenous treatment effect (METE) model. This differentiates the current study from similar studies by Mulwa and Visser (2020), Amare and Simane (2018) and Pangapanga et al. (2012). Mulwa and Visser (2020) used a step-wise error correction model to analyse the effect of adaptation to climate shocks on food security and concluded that, livestock and crop diversification improves food security. Also, Amare and Simane (2018) employed the propensity score matching to examine the effect of climate change adaptation on food security and confirmed the positive effect of adaptation on food security. Using the Tobit regression model, Pangapanga et al. (2012) established that, while flood and drought adaptation strategies such as irrigation enhances food availability, combination of irrigation farming with income generation activities reduces food availability due to labour diversion.

The contribution of this paper is four fold: first, the novelty in this study is the use of multinomial endogenous treatment effect (METE) model to examine the determinants of on-farm and non-farm adaptation strategies and their effects on food security. As previous studies have focused on adapters and non-adapters and have failed to account for differences between adapters that employ on-farm and non-farm strategies as done in this study. Second, the adoption of METE model account for potential endogeneity and selection bias arising from observed and unobserved factors, as previous studies employed methods that failed to account for unobserved factors such as innate skills. Third, while most empirical studies focus on farmers' adaptation decisions in response to climate events on average, this study examine farmers' adaptation decisions to a specific form of weather event, thus flood. Finally, it examines the effect of on-farm and non-farm adaptation strategies on flood resilience of farm household.

The remainder of the study is structured as follows: section two focuses on the methodological issues; section three is on results and discussion of findings; and section four concludes the study with policy implications.

## 2 Materials and methods

### 2.1 Analytical framework and empirical model

The theory underlying this research is the utility maximization theory. The theory posits that, a farmer is a rational economic agent that takes decision based on expected benefits or satisfaction (Kato et al., 2011; Abid et al., 2016). Based on this theory, a farmer makes a rational decision by choosing adaptation strategy that will give him or her, the highest utility. However, a farmer's decision to adapt to flood event is a voluntary self-selection. This implies that a farmer who adapt to flood shock by adopting a particular strategies may be systematically different from those farmers who response using a different strategy or did nothing. For instance, a farmer who adapts to flood event may have some innate skills which is unobservable and may be correlated with the outcome variable, per capita food expenditure. To account for self-selection, observable and unobservable heterogeneity that influences farmers' adaptation decision and food security status, the multinomial endogenous treatment effects (METE) model was employed (Deb and Trivedi, 2006). The METE model is a two-stage model. In the first stage, a mixed multinomial logit was used to model farmers' adaptation decisions and the ordinary least square (OLS) was employed to estimate the effect of each adaptation strategies on food security.

In the first stage, a farmer chooses an adaptation strategy from three mutually exclusive options, namely (0) non-adapter, (1) on-farm strategies and (2) non-farm activities. Based on focus group discussions with the farmers and previous climate adaptation studies, farmers who adapted to flood events were presented with two mutually exclusive adaptation strategies: on-farm and non-farm activities, and were asked to choose one strategy that they rely on most to help them adapt to flood events. Following this question, the farmers were probed further to state the specific adaptation strategies used under selected strategy. The specific on-farm strategies included early planting, upland planting, crop rotation and adoption of early maturing plants. However, petty trading and selling of asset such as livestock were the specific non-farm flood adaptation strategies used by the farmers. Consequently, a farmer, *i*, chooses an adaptation strategy *j* with utility (V_ij_) as oppose to all other strategies, *k*, with utility (*V*_*ik*_) if only if, it maximizes his or her utility. This is expressed as in Eq. (1);

(1) *V*_*ij*_*> V*_*ik,*_*j ≠k.*

Given that the latent utility associated with the *j* strategy is denoted as *FAdapt*∗ then

(2) *FAdapt∗*_*ij*_*= z*_*i*_*α*_*j*_*+λ*_*j*_*l*_*ij*_*+γ*_*ij*_

where *z*_*i*_ is a vector of exogenous covariates such as socio-economic, demographic, farm-level and institutional factors that influence the farmer's adaptation decisions. *α*_*j*_ is the coefficients of *z*_*i*_ and *γ*_*ij*_ are identically distributed error terms. *l*_*ij*_ is a latent factor that incorporate unobservable characteristics common to the farmer's adaptation decisions and outcome variable (i.e food security) such as the farmer's motivation and inherent capabilities (Abdulai and Huffman, 2014) and *λ*_*j*_ is the coefficients of *l*_*ij*_. The latent variable *l*_*ij*_ is assumed to be independent from the error term *γ*_*ij*_.

Given that, *j=0* and *FAdapt∗*_*i0*_ *=0* be the control group, which shows a farmer is a non-adapter. Also, if a farmer's adaptation decisions is defined as a set of binary variables *d*_*i*_ *= (di1, d*_*i2*_*, …, d*_*ij*_*)* representing the observed treatment choice and *l*_*i*_ *= (l*_*i1*_*, l*_*i2*_*, …, l*_*ij*_*)*, then the probability of choosing a particular adaptation strategy, conditional on the latent variables is expressed as;

(3) *Pr (d*_*i*_*| z*_*i*_*, l*_*i*_*)= f (z*_*i*_*α*_*1*_*+λ*_*1*_*l*_*i1,*_*z*_*i*_*α*_*2*_*+λ*_*2*_*l*_*i2*_*,…, z*_*i*_*αJ +λ*_*J*_*l*_*Ij*_*)*

Assuming that *f* exhibits a mixed multinomial logit structure expressed as in Eq. (4)

(4) *Pr (d*_*i*_*| z*_*i*_*, l*_*i*_*)= exp(z*_*i*_*α*_*j*_*+λ*_*j*_*l*_*ij*_*) / 1+Ʃ*^*j*^_*k=1*_*exp (z*_*i*_*α*_*k*_*+λ*_*k*_*l*_*ik*_*)*

Then, in order to normalize the scale for each treatment choice equation, it is assume that each treatment choice is affected by a single latent variable *λ*_*j*_*=1*.

In the second stage, the effect of the farmers' adaptation decisions on food security was estimated. The outcome variable, the natural logarithm of per capita food expenditure (y) was used as indicator for household's food security status. The expected outcome equation for the farm household, *i*, is expressed as in Eq. (5);

(5) *E(y*_*i*_*| d*_*i*_*,x*_*i*_*, l*_*i*_*)= x*_*i*_*δ + Ʃ θ*_*j*_*d*_*ij*_*+Ʃμ*_*j*_*l*_*ij*_

where *x*_*i*_ is a set of exogenous covariates with associated coefficients *δ*; *d*_*ji*_ represent the treatment effects (i.e. on-farm and non-farm strategies) relative to the control choice (non-adapters). Then, *θ*_*j*_ shows the effects of a farmer's adaptation decision on farm household food security. Given that *E(y*_*i*_*| d*_*i*_*,x*_*i*_*, l*_*i*_*)* is a function of latent variable *l*_*ij*_, the outcome is affected by observed factors that also affect the adaptation decisions. A positive (negative) factor loading of parameter *μ*_*j*_ implies that treatment and outcome are positively (negatively) correlated through unobserved characteristics (Manda et al., 2016). Since the outcome variable is continuous, a normal (Gaussian) distribution function was used and the model was estimated using Maximum Simulated Likelihood (MSL) method.

For more robust identification of the model (Deb and Trivedi, 2006), the study used information on flood occurrence as an instrumental or exclusion restriction variable to identify the model. The instrumental variable is a binary variable which takes one if a sampled farmer had information on flood occurrence and zero otherwise. Information on flood occurrence may strongly affect a farmer's choice of adaptation strategies to flood events, but it is unlikely to have a direct effect on farm household food security status. Studies by scholars such as Di Falco and Veronesi (2013) and Manda et al. (2016) have demonstrated that information or training about sustainable agricultural practices can be used as a valid instrumental variable.

Empirically, eqn (4) and eqn(5) are expressed as in Eqs. (6) and (7) respectively.

(6) *Adaptation strategy*_*i*_*= α*_*0*_*+ α*_*1*_*Age + α*_*2*_*Gender + α*_*3*_*Married + α*_*4*_*Education+ α*_*5*_*Household size + α*_*6*_*FBO+ α*_*7*_*Extension+ α*_8_*NADMO + α*_*9*_*Credit+ α*_*10*_*Number of crops planted + α*_*11*_*Farm size+ α*_*12*_*Farm size squared + α*_*13*_*Perceived severity of flood+ α*_*14*_*Information*

(7) *Food security = δ*_*0*_*+ δ*_*1*_*Age + δ*_*2*_*Gender + δ*_*3*_*Married + δ*_*4*_*Education+ + δ*_*5*_*Household size+ δ*_*6*_*FBO+ δ*_*7*_*Credit + δ*_*8*_*Farm size + δ*_*9*_*Farm size square+ θ*_*10*_*On-farm + θ*_*11*_*Non-farm*

The study also examined the factors that influence households' resilience to flood risks. The subjective measure of resilience, where farmers self-evaluate their resilience level was adopted in this study (Jones and Tanner, 2017). Subsequently, the time duration in which farm households' recovered from flood shocks was used as a measure of households' resilience to flood risks. The recovery period adopted include same season (0), after one season (1), after two season (2) and never (3). In this study, household who recovered from flood shocks in the same season have a higher resilience than those who recovered after one season or never. Since the response is ordered, the ordered probit model was used to estimate the determinants of households' resilience. Following Greene (2003), the ordered probit model is expressed as in Eq. (8);

(8) *R*_*i*_*∗ = λ*_*j*_*’X*_*i*_*+ e*_*i*_

where *R*_*i*_*∗* is an unobserved variable representing the recovery period ordered from 0 to 3 associated with household *i*, *X*_*i*_ is a vector of independent variables that affect the recovery periods and *λ* is the vector of parameters to be estimated. Further, *e* is the random error term which exhibits a standard normal distribution.

The observed discrete responses of the outcome variable, *R*_*i*_, is presented as in Eq. (9)

(9) $$\begin{array}{l} R_{i}=\;0\;if\;R_{i}^{*}\leq 0\\ R_{i}=1\;if\;0<R_{i}^{*}\leq 1\\ R_{i}=2\;if\;1<R_{i}^{*}\leq 2\\ R_{i}=3\;if\;R_{i}^{*}>2 \end{array}$$

The cut-off points of each category in Eq. (9) shows the threshold within which the farmers' latent decisions are taken, and is assumed to be monotonically increasing in the value of the latent variable. Consequently, the empirical specification of Eq. (9) is expressed as in Eq. (10);

(10) *Recovery period = λ*_*0*_*+ λ*_*1*_*Age + λ*_*2*_*Age squared + λ*_*3*_*Gender + λ*_*4*_*Education + λ*_*5*_*Income+ λ*_*6*_*Household size + λ*_*7*_*FBO + λ*_*8*_*NADMO+ λ*_*9*_*Credit + λ*_*10*_*Farm size + λ*_*11*_*Perceived severity of flood + λ*_*12*_*Farm_water+ λ*_*13*_*Information + λ*_*14*_*On-farm + λ*_*15*_*Non-farm*

The independent variables adopted for the study was based on previous studies and is presented in Table 2.

### 2.2 Study area and data collection

The study was conducted in Upper East region of Ghana. The region population is rural (79%) and characterised by Guinea and Sudan savannahs (GSS, 2013). It is one of the poorest regions in Ghana that is highly prone to the adverse effects of climate change. Flood occurrence in the district has increase significantly, which is attributed to climate change and the spilling of the Bagre dam in Burkina Faso (Yiran and Stringler, 2016). The region is characterized by a single rainy season, yet, majority of the people engage in agriculture (83.7%) which is mainly rain-fed (GSS, 2013; Yiran and Stringler, 2016).

Multistage sampling technique was used to select the farm households for this study. In the first stage, two districts were randomly selected from thirteen (13) flood prone districts that were affected by the 2018 floods in the Upper East region. These districts were Builsa-North and Kasena-Nankana West districts. Then, three communities each were randomly selected from a list of flood prone communities in the two selected districts, given a total of six communities. The communities were Sandema, Chuchuliga and Wiaga in Builsa-North district and Kayoro, Chiana and Nyangolingo in Kasena-Nankani West district (see Table 1). As presented in Table 1, in the second stage, 30–40 farm households were randomly selected from each selected community, with the number of farm households selected from each community proportional to the size of the number of affected farm households. A total of 230 farm households were selected and interviewed using structured questionnaire.

**Table 1:** Selected communities and sample allocation.

Districts	Communities	Sampled households
Builsa-North	Sandema	40
	Chuchuliga	40
	Wiaga	40
**Sub total**		**120**
Kasena-Nankana West	Kayoro	40
	Chiana	40
	Nyangolingo	30
**Sub total**		**110**
**Total**		**230**

## 3 Results and discussions

### 3.1 Descriptive statistics of key variables

Table 2 presents the descriptive statistics of the key variables of interest. Based on the results, majority of the household heads were male (73%) and were married (80%). The average age of household head was about 46 years. Education level among the sampled household head was low, with an average of six years of formal education. Further, the mean household size was six people. However, the average farm size was approximately 3 acres. Most of the sampled households (61%) had their farms located near water bodies, increasing their vulnerability to floods.

**Table 2:** Descriptive statistics of the sampled households.

Variables	Definition	Measurement	Statistic	
			Mean	Std. Dev.
Food security	Per capita food expenditure (monthly)	Ghana cedis	30.66	33.753
On-farm	On-farm adaptation strategies	1 = if yes; 0 = otherwise	0.59^a^	
Non-farm	Non-farm adaptation strategies	1 = if yes; 0 = otherwise	0.34^a^	
Non-adapters	Non-adapters to flood events	1 = if yes; 0 = otherwise	0.07^a^	
Age	Age of household head	Number of years	45.75	15.92
Gender	Gender of household head	1 = if male; 0 = otherwise	0.73^a^	
Married	Marital status of household head	1 = if yes; 0 = otherwise	0.80^a^	
Education	Years of education of household head	Total number of years of formal education	6.14	6.41
Income	Household total monthly expenditure as a proxy for household income	Ghana cedis	645.58	581.57
Household size	Number of household members	Number of household members	6.30	3.60
FBO	Member of farmer-based organization (FBO)	1 = if yes; 0 = otherwise	0.20^a^	
Extension	Access to extension services	1 = if yes; 0 = otherwise	0.26^a^	
NADMO	Access to National Disaster Management Organization (NADMO) services	1 = if yes; 0 = otherwise	0.25^a^	
Credit	Access to credit	1 = if yes; 0 = otherwise	0.21^a^	
Number of crops planted	Total number of crops planted	Number of crops planted	2.68	0.72
Farm size	Farm size	Acres	2.70	1.26
Perceived severity of flood	Perceived severity of flood events over the past ten years	1 = if yes; 0 = otherwise	0.61^a^	
Farm_water	Farm near to water body	1 = if yes; 0 = otherwise	0.61^a^	
Information	Received information on flood events	1 = if yes; 0 = otherwise	0.58^a^	

NB: *a* is the percentage for the dummy variables.

Access to credit has been recognized as crucial in enhancing farmers' capability to adapt to climate related shock such as flood events. Based on the results, access to credit among the sampled households was low as only 20% of the farmers had access. In addition, about 20% of the sampled households belonged to FBOs, 25% had access to National Disaster Management Organization (NADMO) services and received relief items. Further, 26% of the sampled households had access to extension services and most (58%) had received information on flood occurrence. On average, household's monthly per capita food consumption expenditure was about GHS 31.00 cedis. Similarly, the average monthly expenditure (used as a measure for household income) was approximately GHS 646 cedis.

### 3.2 Perception of flood occurrence, flood adaptation strategies and perceived causes

Farm households' perception of flood occurrence has implications on adaptation decisions. As shown in Table 2, on average, most of the sampled households had perceived flood to be severe (61%). To reduce the adverse effects of flood events, farmers adopted various adaptation strategies which were categorized into two mutually exclusive groups in this study. Based on the results, approximately 59% adopted on-farm practices, 34% engaged in non-farm activities and 7% were non-adapters (see Table 2).

Table 3 presents the perceived causes of flood by farm households. To the sampled households, the causes of flood include heavy downpours (40%), and spillage of the Bagre dam by neighbouring Burkina Faso (26%). A few attributed flooding to environmental degradation (5%) and about 29% of the farmers' indicated that all the enlisted three (3) factors caused flooding in the study area.

**Table 3:** Farmers' perception of causes of flood (in percentage).

Causes of flood	Percentage (%)
Heavy downpours	39.57
Spillage of the Bagre dam	25.65
Environmental degradation	4.78
All the above	28.70
None of the above	1.30
Total	100

### 3.3 Adaptation strategies and recovery period from flood shocks

Table 4, present a cross tabulation between farmers' recovery period and adaptation strategies. The chi-square test reveals that there is no correlation between recovery period and adaptation strategies. Even though there are no significant differences between the adaptation strategies used and recovery period from flood events, it is necessary to discuss the frequency distribution. From the results, farmers' who engaged in non-farm activities (24.36%) were more likely to recover faster in the same season than those who adopted on-farm strategies (22.96%) and non-adapters (5.88%).

**Table 4:** Recovery periods and adaptation strategies to flood events (%).

Response	Adaptation strategies			Total
	Non-adapters	On-farm	Non-farm	
Same season	5.88	22.96	24.36	22.17
After one season	35.29	43.70	30.77	38.70
After two season	47.06	23.70	29.49	27.39
Never	11.76	9.63	15.39	11.74
Total	100	100	100	100
Pearsonchi2 (6)	9.024			

### 3.4 Drivers of on-farm and non-farm flood adaptation strategies

Table 5 presents the first stage of the multinomial endogenous treatment effects model where a mixed multinomial logit regression was used to estimate the determinants of on-farm and non-farm flood adaptation strategies. The Wald test shows that, the null hypothesis is rejected at 1% level, suggesting that all the regression coefficients are jointly statistically significant; thus the model fits the data.

**Table 5:** Mixed Multinomial logit estimates of determinants of on-farm and non-farm flood adaptation strategies.

Variable	Adaptation strategies to flood events			
	On-farm		Non-farm	
	Coeff.	Std. err	Coeff.	Std. err
Age	-0.056∗∗∗	0.020	-0.016	0.021
Gender	0.240	0.667	-0.737	0.673
Married	0.931	0.623	1.212∗	0.671
Education	-0.099∗∗	0.049	-0.105∗∗	0.053
Household size	0.019	0.100	-0.083	0.112
FBO	0.504	0.689	-1.236	0.793
Extension	2.134∗∗	0.882	1.465	0.944
NADMO	0.704	0.812	-0.137	0.827
Credit	-1.489∗∗	0.724	-1.160	0.717
Number of crops planted	-0.279	0.484	-0.500	0.501
Farm size	-1.854∗	0.993	-1.749∗	0.995
Farm size squared	0.405∗∗	0.173	0.398∗∗	0.170
Perceived severity	0.355	0.583	-0.281	0.595
Information	1.481∗∗	0.725	1.377∗	0.739
Constant	5.597∗∗	2.486	5.560∗∗	2.478
Observations	230			
Chi square	257.74∗∗∗			
Log pseudolikelihood	-484.636			

∗∗∗, ∗∗ and ∗ shows that it is statistically significant at 1%, 5% and 10% level respectively.

Based on the perceived severity of flood impacts, farm households adopted a variety of adaptation strategies to reduce the adverse effects of flood impacts on their livelihood. The adaptation strategies were categorized into on-farm and non-farm strategies to explore the drivers of these strategies. The determinants of on-farm and non-farm flood adaptation strategies are presented and discussed as follows. The coefficient of age was negative and significantly affects on-farm adaptation decision only. The negative significant effect of age on decision to adopt on-farm practices suggests that, a year increase in the age of the farmer decreased the probability of adopting on-farm strategies. The result is conceivable since younger farmers are more likely to be exposed to new technology and innovative, and less risk averse to adopt modern farm technologies. This result is in tandem with the findings of Ali and Erenstein (2017) who reported that younger farmers are more innovative and keen to try new technology to enhance crop yield. Education also plays a part in the decision of a farmer to adopt both on-farm and non-farm strategies in response to flood shocks. Surprisingly, education decreases the probabilities of a farmer adopting on-farm practices and engaging in non-farm activity. It is expected that education will provides skills and knowledge required to adopt modern farm technology and secure a job other than farming. This observation is partly attributed to the fact that, educated farmers, generally engage in farming on a part time basis while they commit to their full-time job. Further, since petty trading was the major non-farm activities engaged in by farmers, they do not require a special skill and knowledge. For these reasons the educated farmers are less likely to adopt on-farm and non-farm flood adaptation strategies. These results contradict the findings of other studies (e.g. Abid et al., 2016; Deressa et al., 2009).

The coefficient of marital status was significant and positively related to farmers' decision to participate in non-farm activities only. Generally, married households are associated with larger family size with associated increase consumption expenditure. To provide for the households, a farmer engaged in non-farm activities to ease capital constraint and smoothen basic consumption expenditure of the family. As expected, access to extension had a positive significant effect on the decision to adopt on-farm strategies only, reflecting the role of information which empowers farmers to adopt modern technology. Among other things, extension officers are required to provide information and educate farmers on improved farm technologies and also connect farmers to input-sellers. This information empowers the farmer to take a well informed decision in choosing the appropriate on-farm strategies. The result is strongly supported by Abid et al. (2016) and Deressa et al. (2009).

Surprisingly, access to credit had a negative effect on on-farm flood adaptation strategy but not on non-farm activity. The negative significant effect of access to credit on a farmer's decision to adapt suggests that, a farmer who had access to credit was less likely to use on-farm strategies, contradicting the expectation of the research. The expectation was, access to credit makes farmers more financially resourced to adopt improved agricultural technology that enable them cope with flood events. The possible explanation for this occurrence is that, farmers may use credit for consumption rather than investing in farm inputs since the flood shock experienced could deplete their resources. The observation of the result does not suggest that the provision of credit to farmers should be discouraged but, rather, it calls for regulation of credit to ensure effective use of it. In other studies, Ali and Erenstein (2017) and Alhassan et al. (2019) reported that access to credit encourage farmers' to adapt better to climate change.

Farm size and the square of farm size respectively had a negative and positive relationship with adaptation related to on-farm and non-farm strategies. This suggests that, an increase in farm size decreases farmers' adaptation to flood events until a certain turning point beyond which adaptation increases with farm size. Thus, the relationship between farm size and adaptation decisions related to on-farm and non-farm activity exhibits a U-shaped. This indicates that farmers with larger farm size face lesser restrictions to adapt to flood events; which is in line with the generally reported positive effect of farm size on adaptation to climate change. Larger farm size reflect the role of wealth/asset in the adoption of on-farm strategies as higher level of wealth means a greater capacity by the farmer to finance on-farm strategies. Hence, provides the opportunity for farmers to experiment and invest in new technologies (Ali and Erenstein, 2017). Further, more resourced and endowed farmers have the capacity to invest in non-farm activities and take the associated risk of investing in other business opportunities. These results confirmed the findings of Sahu and Mishra (2013) and Abid et al. (2016) who established a positive association between farm size and adaptation to climate change.

Consistent with expectation, access to flood information had positive and significant effect on farmers' decision to adapt using both on-farm and non-farm strategies. Provision of appropriate information and timely advice to farmers on various measures that could be taken to reduce the adverse effect of flooding empowers the farmers to make better decisions. Further, information available to farmers prior to and during the flood makes them certain about the risks and thus empowers them to adapt to flood events. This result confirmed the findings of Thennakoon et al. (2020) who concluded that access to early warning information empower farmers to adapt to climate change.

### 3.5 Effect of on-farm and non-farm flood adaptation strategies on food security

Table 6, presents the second stage estimates of the METE model, which shows the effect of on-farm and non-farm flood adaptation strategies on food security. The coefficient of lambda (non-farm strategy) is positive and significant at 5% level, suggesting that there is a positive selection bias. This support the hypothesis that, self-selection is present, justifying the use of the METE technique. Further the positive sign, demonstrate that the unobservable characteristics that increase the likelihood of farm household's choosing non-farm activities as adaptation strategy to flood events are associated with a higher farm household food security status than what could be expected when this adaptation strategy was randomly assigned to the farm households.

**Table 6:** Average treatment effect of on-farm and non-farm flood adaptation strategies on farm household food security.

Variable	Per capita food expenditure (ln)	
	Coeff.	Std. err
On-farm	0.212∗∗∗	0.075
Non-farm	0.170∗∗	0.078
Age	0.001	0.002
Gender	0.053	0.048
Married	0.029	0.053
Education	0.013∗∗∗	0.003
Household size	-0.065∗∗∗	0.010
FBO	0.078∗	0.042
Credit	0.049	0.046
Farm size	-0.009	0.066
Farm size squared	0.001	0.011
Constant	-1.105∗∗∗	0.285
/lnalpha	2.202∗∗∗	0.191
/lambda_category2 (on-farm)	-0.011	0.009
/lambda category3 (non-farm)	0.014∗∗	0.007
Alpha	9.046	1.730

The baseline category of flood adaptation strategy is non-adaptors. The estimates are based on 500 simulations draw per observation based on a Halton sequence. ∗∗∗ significant at 1%; ∗∗ significant at 5% and ∗significant at 10%.

The main interest of this research was to examine how farmers' adaptation decisions affect food security and the results met the expectation of the research. The findings demonstrated that adapting to flood events using both on-farm and non-farm strategies had positive significant effect on per capita food expenditure. This suggests that adaptation related to on-farm and non-farm activities are indeed effective strategies since they enhances farm household food security situation. On-farm practices such as adoption of improved farm technology leads to higher productivity which enhances households' food security. As majority of the farmers in the study area depend largely on their own farm produce for household food consumption. The result agrees with previous research by Abid et al. (2016) who showed that adaptation to climate-related shocks enhances food security. On non-farm activities, the result supports the widely accepted view that non-farm income improves households' food security. As reported by Owusu et al. (2011), farmers engaged in non-farm activities to supplement own production with food purchased from the market. Income from non-farm activities increase household average income, their consumption expenditure, which enable them better cope to flood shock. Gulseven (2014) also reported that non-farm income enhances farm households' welfare.

With the other control variables, education had a positive significant effect on food security, suggesting that a year increase in farmers' educational level enhance household food security status. Formal education enhances the opportunity of farmers to have assured and steady flow of income through salaried job, which improves and smoothen households' food consumption expenditure. As in previous studies Mango et al. (2014) and Bashir et al. (2013) supported the argument that education enhances food security. Expectedly, household size had negative significant effect on food security, implying that, an increase in household members, decreased food security status of household. This is reasonable as larger households imply more people to feed, thereby putting pressure on consumption. This supports the findings of Oduniyi and Tekana (2020). Also, it was observed that, farmers who belong to FBO were more food secured than those who were not members of FBO. This is plausible and meets a priori expectation of the research, as FBO provide information and resources that enhances farmers understanding of different management methods and opportunities they can engage in to adapt better to flood shocks. The positive effect of FBO on farm households' food security status suggest that addressing capital, input and output market inefficiencies via farmer based groups can enhance the welfare of the rural poor farmers.

### 3.6 Determinants of flood shocks recovery period of farm households

The study also examines the determinants of farm households' recovery period to flood shocks. Table 7, presents the drivers of farm households' flood recovery period (used as indicator for farm households' resilience). The estimated cut-off-points cut1, /cut2, and /cut3 shows the lower and upper threshold of the model. Based on the results presented, the lower and upper threshold is -2.527 and -0.347 respectively. This implies that the cut-off points are monotonically increasing and thus, as the latent variable rise, the observed outcomes never decrease. Also the cut-off-points (cut1,/cut2, and/cut3) represent the respective intercept of each function of the dependent variable, which ranged from same season (0), after one season (1), after two season (2) and never (3). Therefore, the negative coefficients suggest that an increase in the independent variables means farm households' recovered faster from flood shocks.

**Table 7:** Ordered probit estimates of determinants of flood shocks recovery period of farm households.

Variable	Coeff.	Std. Err	Same season		After one season		After two season		Never recovery	
			Marginal effect	Std. Err.	Marginal effect	Std. Err.	Marginal effect	Std. Err.	Marginal effect	Std. Err.
Age	-0.056∗∗	0.024	0.015∗∗	0.006	0.004∗∗	0.002	-0.009∗∗	0.004	-0.010∗∗	0.004
Age squared	0.001∗∗	0.0002	-0.0001∗∗	0.00006	-0.00004∗∗	0.00002	0.00009∗∗	0.00004	0.00010∗∗	0.00004
Gender	-0.048	0.170	0.013	0.045	0.004	0.013	-0.008	0.028	-0.009	0.031
Education	-0.026∗	0.015	0.007∗	0.004	0.002	0.001	-0.004∗	0.003	-0.005∗	0.003
Income	0.0001	0.0002	-0.000	0.000	0.000	0.000	0.000	0.000	0.000	0.000
Household size	0.006	0.031	-0.002	0.008	-0.0005	0.002	0.001	0.005	0.001	0.006
FBO	-0.502∗∗∗	0.195	0.132∗∗∗	0.050	0.039∗∗	0.019	-0.082∗∗∗	0.032	-0.090∗∗∗	0.037
NADMO	0.286	0.179	-0.075	0.047	-0.022	0.015	0.046	0.029	0.051	0.032
Credit	-0.087	0.182	0.023	0.048	0.007	0.014	-0.014	0.030	-0.015	0.033
Farm size	0.018	0.062	-0.005	0.016	-0.001	0.005	0.003	0.010	0.003	0.011
Perceived severity of flood	0.586∗∗∗	0.157	-0.154∗∗∗	0.040	-0.046∗∗∗	0.017	0.095∗∗∗	0.025	0.105∗∗∗	0.031
Farm_ water	-0.206	0.154	0.054	0.040	0.016	0.013	-0.034	0.025	-0.037	0.028
Information	0.110	0.162	-0.029	0.043	-0.009	0.013	0.018	0.026	0.020	0.029
On-farm	-0.666∗∗	0.288	0.175∗∗	0.076	0.052∗∗	0.026	-0.108∗∗	0.047	0.119∗∗	0.053
Non-farm	-0.521∗	0.296	0.137∗	0.078	0.041∗	0.025	-0.085∗	0.048	-0.093∗	0.054
/cut1	-2.527	0.718								
/cut2	-1.349	0.707								
/cut3	-0.347	0.707								
Observations	230									
Chi square	46.05∗∗∗									
Log likelihood	-277.716									
Pseudo R2	0.077									

Note: ∗∗∗, ∗∗ and ∗ indicates significance at 1%, 5% and 10%, respectively.

Of particular interest to this study is the effect of adaptation strategies on farm households' recovery period, which indicates that farm households' recovered faster when they adapt to flood events. Specifically, farmers who employed on-farm strategies recovered on the same season from flood shocks. On-farm strategies such as the adoption of modern technology have the potential to reduce crop failure and increase yield, making households' more resilience to flood shocks. The positive effect of on-farm practices on flood recovery period corroborates the finding of Jiri et al. (2017). Similarly, engagement in non-farm activities enhance the recovery period of farmers from flood shocks. As the results indicated, farmers who engaged in non-farm activities recovered within the same period. This is reasonable as income from non-farm activities increase farm households' average income and smoothen consumption which buffers them against shocks.

On the other determinants of flood recovery period, the results showed that, a year increase in educational level increases the likelihood of recovering within the same period by 0.007 and decreases the farm households' probability of never recovering from flood shock by 0.005. This is expected as formal education endows people with the capability to use information and resources to cope, thereby increasing the educated households' resilience to flood shocks. The effect of age and age square on recovery period is negative and positive respectively, suggesting that older headed households recovered faster from flood shocks than younger headed households. Generally, the elderly typically have control over resources and the power to make decisions than younger ones; which increases their capabilities to cope and recover faster from flood shocks. This confirmed the findings of Adzawla et al. (2019) who reported that older farmers were more likely to recover faster from climate shock because they were more resourced but contradicts that of Jiri et al. (2017).

The negative significant effect of FBO on recovery period, suggests that farmers who belonged to farmer groups were more likely to recover from flood shocks within the same period than those who do not belonged to FBO. Farmer based organization is a very important social network which provides farmers with information and resources that enhance their resilience to flood shock. Surprisingly, farmers who perceived flood to be severe did not recovered fast, but were more likely to recover after two seasons or never recovered from flood shocks. It was expected that farmers who perceived flood to be severe would be able to adapt better and, thus improve their resilience to flood shock. However, this result is in line with the finding of Adzawla et al. (2019).

## 4 Conclusion and policy implications

This study adopts a multinomial endogenous treatment effect (METE) model to examine the effect of flood adaptation strategies on farm households' food security. Besides, the ordered probit model was used to examine the drivers of households' recovery period from flood shocks. Data was collected from 230 farm households in the Upper East region, Ghana. Farmers employed on-farm and non-farm activities as flood adaptation strategies. Estimation results confirmed the view that adaptation decisions related to both on-farm and non-farm strategies improve households' food security and significantly enhance their recovery period from flood shocks. Determinants of farmers' adaptation decision related to on-farm practices include age, education, access to extension, credit, farm size and information on flood occurrence. The factors that affect non-farm flood adaptation strategy were education, married household heads, farm size and information on flood occurrence. Other factors that significantly influence the recovery period of farmers were age, education, FBO and perceived severity of flood. In conclusion, the study validated the widely held view that adaptation to flood events through on-farm practices and non-farm activities improve food security outcomes in addition to making farm households' recover faster from flood shocks.

The findings from this study have important policy implications. The study highlights the benefits of social network, extension services and information on flood occurrence to adaptation decisions. Thus, providing information on flood occurrence and adaptation strategies available to farmers may help them adapt better and thus build their resilience to flood shocks. It is important that the Ministry of Food and Agriculture intensifies and promote extension services in rural areas. Further, the results established the importance of on-farm and non-farm activities in enhancing food security and building resilience of farm households in flood prone areas. Enhancing the endowments of farmers through access to extension services and alternative livelihoods has the potential to improve their food security. Government can use her flagship programs, Planting for Food and Jobs (2017–2022), and One District, One Factory (1D1F) as potential avenue to provide farm households the opportunity to access improved agricultural inputs and non-farm work.

## Declarations

### Author contribution statement

Hamdiyah Alhassan: Conceived and designed the experiments; Performed the experiments; Analyzed and interpreted the data; Contributed reagents, materials, analysis tools or data; Wrote the paper.

### Funding statement

This research did not receive any specific grant from funding agencies in the public, commercial, or not-for-profit sectors.

### Competing interest statement

The authors declare no conflict of interest.

### Additional information

No additional information is available for this paper.
